# miRNA arm selection and isomiR distribution in gastric cancer

**DOI:** 10.1186/1471-2164-13-S1-S13

**Published:** 2012-01-17

**Authors:** Sung-Chou Li, Yu-Lun Liao, Meng-Ru Ho, Kuo-Wang Tsai, Chun-Hung Lai, Wen-chang Lin

**Affiliations:** 1 Genomics Research Center, Academia Sinica, Taipei, Taiwan; 2 Institute of Biomedical Informatics, National Yang-Ming University, Taipei, Taiwan; 3 Institute of Biomedical Sciences, Academia Sinica, Taipei, Taiwan; 4 Department of Medical Education and Research, Kaohsiung Veterans General Hospital, Kaohsiung, Taiwan

## Abstract

**Background:**

MicroRNAs (miRNAs) are small non-protein-coding RNAs. miRNA genes need several biogenesis steps to form function miRNAs. However, the precise mechanism and biology involved in the mature miRNA molecules are not clearly investigated. In this study, we conducted in-depth analyses to examine the arm selection and isomiRs using NGS platform.

**Methods:**

We sequenced small RNAs from one pair of normal and gastric tumor tissues with Solexa platform. By analyzing the NGS data, we quantified the expression profiles of miRNAs and isomiRs in gastric tissues. Then, we measured the expression ratios of 5p arm to 3p arm of the same pre-miRNAs. And, we used Kolmogorov-Smirnov (KS) test to examine isomiR pattern difference between tissues.

**Results:**

Our result showed the 5p arm and 3p arm miRNA derived from the same pre-miRNAs have different tissue expression preference, one preferred normal tissue and the other preferred tumor tissue, which strongly implied that there could be other mechanism controlling mature miRNA selection in addition to the known hydrogen-bonding selection rule. Furthermore, by using the KS test, we demonstrated that some isomiR types preferentially occur in normal gastric tissue but other types prefer tumor gastric tissue.

**Conclusions:**

Arm selections and isomiR patterns are significantly varied in human cancers by using deep sequencing NGS data. Our results provided a novel research topic in miRNA regulation study. With advanced bioinformatics and molecular biology studies, more robust conclusions and insight into miRNA regulation can be achieved in the near future.

## Background

MicroRNAs (miRNAs) are small non-protein-coding RNAs. Their final functional products are RNA molecules rather than proteins. Although the functional products are different, like many protein-coding genes, miRNA genes also need several maturation steps to form the functional products, single-strand RNAs with approximately 22 nt. in length. After miRNA genes are transcribed, the full-length transcripts (pri-miRNAs) form a hairpin structure (pre-miRNA) plus two un-paired tails, which are trimmed out by Drosha. The pre-miRNA, composed of 5p arm, 3p arm and terminal loop, is further processed by Dicer, trimming out the terminal loop and releasing the miRNA/miRNA* duplex. The miRNA/miRNA* duplex is subsequently processed by RISC, which unwinds the miRNA/miRNA* duplex at the end with weaker hydrogen binding. So, the strand with free 5' end is selectively included into RISC and served as mature miRNA [1,2].

Although mature miRNAs are derived from full-length transcripts of miRNA genes, the expression of miRNA genes does not guarantee the expression of mature miRNA. In other words, not all of the pri-miRNAs are processed into mature miRNAs [3-5]. This unequal maturation control comes from several regulatory steps. First, Drosha and Dicer have higher affinity to the pri-miRNAs and pre-miRNAs, respectively, whose terminal loops are moderate in size [6,7]. Furthermore, longer stem (~33 bp) of pri-miRNA is preferred by Drosha [6]. Second, owing to the hydrogen-bonding selection mechanism, the 5p arm and 3p arm of the same pre-miRNA usually have unequal likelihoods to be selected as mature miRNAs [8].

Up to now, this hydrogen-bonding-based selection rule seems to be the major view point. However, recent studies brought new concepts that challenged the traditional miRNA maturation mechanism. First, previous studies showed that the orthologous pre-miRNAs, although highly similar with each other, preferred the 5p arm in one species but the 3p arm in another species [9,10]. This result challenged the hydrogen-bonding selection rule, implying that there could be other regulation mechanism controlling the 5p arm or 3p arm selection. Second, with the application of NGS technology, mature miRNAs were often observed to present as different isoforms, named isomiRs [9,11,12]. Further analysis has implied that different isomiRs may contribute to regulations in *Drosophila *development [13].

In this study, we conducted in-depth analyses on these issues by using NGS technology to quantify the expression profiles of miRNAs and isomiRs in human gastric tissues. By measuring the expression ratios of 5p arm to 3p arm between tissues, we showed the 5p arm and 3p arm miRNA derived from the same pre-miRNAs have different tissue expression preference, one preferred normal tissue and the other preferred tumor tissue, which strongly implied that there could be other mechanism controlling arm selection in addition to the hydrogen-bonding selection rule. Furthermore, by using the Kolmogorov-Smirnov statistics test, we demonstrated that some isomiR types preferentially occur in normal gastric tissue but other types might prefer tumor gastric tissue.

## Methods

### Collecting sequence reads

We applied Illumina (Solexa) platform for small RNA sequencing. One pair of normal gastric tissue (G1245N) and gastric tumor tissue (G1245T) were lysed with TissueLyser (Qiagen), followed by RNA extraction with TRIzol reagent (Invitrogen) according to the manufacturer's protocol. Then, the RNA samples were processed and sequenced. The generated sequence reads were processed to remove the 3' end adapter, if applicable. Only the clean reads, reads with adapter detected and trimmed, were used for analysis. Besides, considering the length distribution of mature miRNAs, we selected only the clean reads with length 18 to 25 nucleotides for analysis.

The initially analyzed normal and tumor data sets are not equal in size (19.7 million reads in G1245N and 26.0 million reads in G1245T). Therefore, we tried to normalize them with a regression model. As a result, we got the equation *y *= 1.3004*x*-0.1457, where x and y denote the expression levels of miRNAs in G1245N and G1245T library, respectively. After this normalization procedure and plotted in a scatter plot, most of the data points distributed near the line with the slope of 0.9874 and the R^2 ^value was 0.8831. This result showed that the expression levels of most miRNAs did not vary between tissues and the miRNA expression data from the two the libraries was comparable.

### Mapping criteria

The clean reads were grouped into unique clean reads, followed by tabulating the count of each unique clean read. For higher confidence, only the unique reads with read count equal to or larger than two were used for mapping back to human pre-miRNAs (miRBase 16). In order to eliminate ambiguous mapped loci caused by the high similarity between human paralogous mature miRNAs, such as hsa-miR-548a and hsa-miR-548b, we allowed no mismatch at the mapping procedure. Previous reports observed nucleotide additions at the 3' end of miRNAs [12,15-18], which may cause mismatches at the mapping procedure. Therefore, using Fernandez-Valverde's strategy [13], we trimmed the last 3' end mismatch one by one until the mapping perfect-match reads are at least 18 nucleotides in length.

### Excluding random matches

With the application of NGS technology, miRNA are reported to exist as isomiRs [9,11,12]. As shown in Additional file 1, the isomiRs (the red line alignments) shift from their corresponding miRBase reference miRNAs (dark and light blue bars) in terms of location. When sequence reads were mapped back to mature miRNAs, the alignment shift may result in mismatches. Therefore, in addition to the perfect match constraint, we adopted an alternative procedure. In order to exclude random match, the difference in start position between mature miRNA and mapped reads must be equal to or less than two nucleotides. While, the difference in end position between mature miRNA and mapped reads must be equal to or less than five nucleotides.

### Experimental validation of miRNA with stem-loop RT-PCR

In this study, we used stem-loop RT-PCR to validate the 5p arm miRNA of hsa-mir-1307 as described previously [20]. The RT primer (CTCAACTGGTGTCGTGGAGTCGGCAATTCAGTTGAGagccgg) contains a stem-loop sequence and a 6-nt overhang sequence resulting in the binding specificity to mature miRNA. For each RT reaction, 1 g of total RNA was converted into cDNA (miRNA-specific stem-loop RT, 2 nM, 500M dNTP and 0.5l Superscript III, Invitrogen, Carlsbad, CA) and was performed as follows: 16°C for 30 min, followed by 50 cycles at 20 °C for 30 s, 42 °C for 30 s and 50 °C for 1 s. Expression of the miRNA was detected with real-time quantitative PCR (RT-qPCR) by the SYBR Green I protocol (Applied Biosystems, Foster City, CA), 200 nM miRNA-specific forward primer (CGGCGGtcgaccggacctcgac), and 200 nM universal reverse primer. RT-qPCR was performed with the following conditions: 95 °C for 10 min, 95 °C for 15 s and 63 °C for 32 s by 40 cycles. All values were normalized against U6 RNA.

### Comparing isomiR patterns between tissues

**Figure 1:**
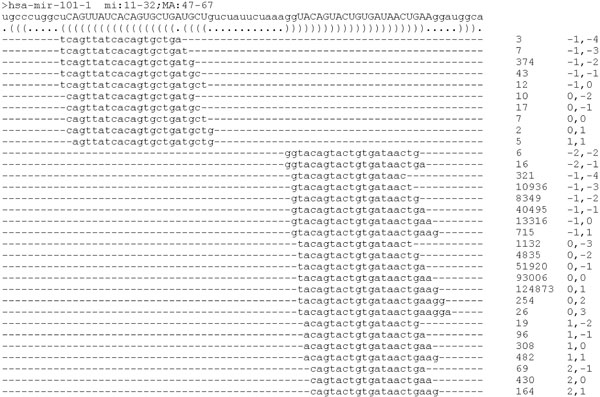
**Mapping result of hsa-mir-101-1 in G1245N library.** hsa-mir-101-1 encode minor miRNA (hsa-miR-101*) at the 5p arm, ranging from nt. 11 to 32 of pre-miRNA, and major miRNA (hsa-miR-101) at the 3p arm, ranging from nt. 47 to 67. The 5p and 3p miRNAs are presented in uppercase and they individually have 10 and 22 isomiR types, each of which is labeled with read count (before normalization) and location offset. The expression level of a mature miRNA is determined by summing the read counts of all its isomiRs.

In this study, we applied the Kolmogorov-Smirnov (KS) test for determining whether isomiR distribution patterns differ between normal and tumor tissue. KS test tries to determine if two datasets differ significantly and has the advantage of making no assumption about the distribution of data. For each mature miRNA, we enumerated its isomiRs in normal and tumor tissue (Figure 1), forming a union, followed by summarizing the read counts of all isomiRs as the expression abundance of the miRNA. Then we assigned the isomiRs with a type ID according to their locations. Next, we generated a table of relative abundance percentage of each isomiR type. By using this table, we may transform the × axis of KS test from numerical data into categorical data (isomiR type). As a result, we may examine individual isomiR type and whether the isomiR distributions are different between normal and tumor tissue according to the generated p-value of KS test.

## Results and discussion

### Summary of sequence reads

In this study, we generated sequence reads of small RNAs from normal gastric tissue (G1245N) and gastric tumor tissue (G1245T). Totally, 32.1 and 32.4million sequence reads were initially collected from G1245N and G1245T library, respectively. After trimming adapter procedure, we collected 23.8 and 29.9 million reads individually in G1245N and G1245T library for further analysis. Further filtered with length and read count criteria, in G1245N and G1245T library, 19.7 and 26.0 million reads were finally used to quantify miRNA expression level.

### Analysis of detected miRNAs

Under our mapping criteria described in Methods, totally there were 17,492,248 and 23,974,839 reads, individually from G1245N and G1245T library, mapped to known human miRNAs (Table 1). Therefore, 88.8% reads in G1245N library belong to miRNA; while, 92.4% reads in G1245T library do so. As mentioned in the previous study [12], the rest non-miRNA reads could be other small non-protein-coding RNAs or degradation product of mRNA. As shown in Table 1, 688 and 705 out of 1,223 known mature miRNAs were detected in G1245N and G1245T library, respectively. Our data is comparable to most NGS studies.

**Table 1:** Summary of analysis on miRNA reads

	G1245N	G1245T
**# Reads**	17,492,248	23,974,839
**# detected pre-miRNAs**^a^	542	556
**# detected miRNAs**^a^	688	705
**# miRNAs at opposite arm**^b^	87	96
**# isomiRs**	5,132	5,305
**# isomiRs with length equal to miRNA**	542	569

We mapped the sequence reads back to human pre-miRNA, quantifying the expression levels of pre-miRNAs, mature miRNA and isomiRs.

^a^: The human pre-miRNA and mature miRNA are based on the definition of miRBase 16. Totally, there are 1,048 pre-miRNAs and 1,223 mature miRNAs, resulting in 1,366 miRNA/pre-miRNA pairs.

^b^: According to miRBase 16, 730 out of 1,048 human pre-miRNAs encode only one mature miRNA at one arm, either 5p or 3p arm. With the improvement on NGS's sequencing intensity, we detected additional opposite-arm mature miRNAs at the pre-miRNAs originally encoding only one miRNA.

We arranged the miRNA reads within the mapped pre-miRNAs. As shown in Figure 1, hsa-mir-101-1 encodes mature miRNAs at both arms. The integer digits in middle column denote the read count of each isomiR. The presentations in the right column denote the location offset relative to reference miRNA annotated with miRBase. So, the reads with presentation "0,0" are exactly the same with reference miRNAs. Examining the counts of all reads, it is not guaranteed that the reference miRNAs from miRBase are the most abundant ones, which was also observed by other studies [11,12,14]. The mapping result of all pre-miRNAs in G1245N and G1245T library can be accessed in Additional file 2 and 3.

### Detection of additional miRNAs at the opposite arms

**Figure 2:**
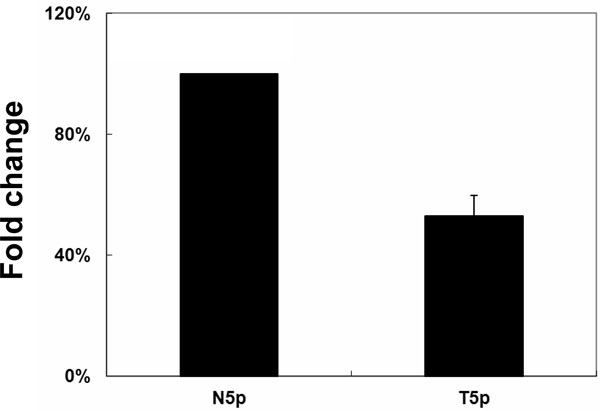
**Detection of 5p arm miRNA of hsa-mir-1307.** According to miRBase annotation, hsa-mir-1307 encodes mature miRNA at only its 3p arm. We used stem-loop RT-PCR to succeed in detecting the additional opposite-arm miRNA in both G1245N and G1245T tissue.

Among 1,048 reported human pre-miRNAs, 730 were reported to encode mature miRNAs at only one arm (miRBase 16 release). However, with the improvement in sequencing depth of NGS platforms, more miRNAs can be also detected at the opposite arms. As illustrated in Additional file 2, hsa-mir-1307 encodes mature miRNA at only its 3p arm by miRBase. However, we detected additional mature miRNA at the opposite arm, the 5p arm, of hsa-mir-1307. In order to confirm the existence of the 5p miRNA of hsa-mir-1307, we designed specific stem-loop RT-PCR assay for validation. At the 5p arm of hsa-mir-1307, eight isomiRs were detected and we designed specific primers to validate the most abundant one. As shown in Figure 2, we can specifically detect the 5p arm miRNA of hsa-mir-1307 in both normal and tumor tissue.

As described in previous study [14], the additional opposite-arm miRNAs are not necessary to be at lower expression levels than the original ones. Many of the additional opposite-arm miRNAs have higher expression level than the original one (Additional file 2 and 3), which might be different from the previous nomenclature rules. In this study, we totally detected 24 additional opposite-arm miRNAs exclusively in G1245N, 33 ones exclusively in G1245T and 63 ones both in G1245N and G1245T library. The discovery of additional opposite-arm miRNAs is because we mapped the sequence reads back to pre-miRNAs rather than only to mature miRNAs. Our study here provides a new way to further interrogate the miRNA/isomiR expression by carefully examining NGS data.

### Analysis of addition fragments generated by 3' end modification

Owing to 3' end modification [12,15-18], the altered nucleotides at the 3' end of reads may cause mismatches at the mapping procedure, making the originally perfect match reads fail to be mapped back to miRNAs. Therefore, we trimmed the terminal 3' end mismatch one nucleotide by one nucleotide, followed by analyzing the trimmed fragments. As a result, 2,766,852 reads in G1245N tissue and 3,319,704 reads in G1245T tissue were found to have nucleotide added at their 3' ends, individually accounting for 14.0% and 12.8% of read collection used for mapping. Without this alternative mapping method, these 14.0% and 12.8% more sequence reads can not be mapped back to human pre-miRNAs, which demonstrates the effectiveness of our alternative mapping procedure.

Previous large-scale investigation on *Arabidopsis thaliana *and *Oryza sativa *demonstrated uridine additions at the 3' end of miRNAs [17]. In this study, we totally observed 499 and 409 kinds of 3' end addition fragments in G1245N and G1245T library, respectively. In addition to uridine addition, we also observed adenine additions. As shown in Table 2, the most and the secondary abundant addition event in G1245N library is adenine and uridine addition, respectively. However, in G1245T library, the two addition events exchanged in terms of abundance. It needs more data to conclude whether such difference is functionally related to biological difference. In summary, adenine and uridine additions account for almost 80% of all addition events. Besides, AA, UA, UU, AU, G and AAA also account for at least 1% of addition events.

**Table 2:** Distribution of the 3' end addition fragments in G1245N and G1245T library

Fragment sequence	Abun. in G1245N (%)	Abun. in G1245T (%)
**A**	50.93	33.25
**U**	30.72	44.07
**AA**	3.39	4.67
**UA**	2.28	4.19
**UU**	2.16	3.12
**AU**	1.53	1.23
**G**	1.40	1.80
**AAA**	1.07	2.10

We analyzed the sequence and the relative abundance of 3' end addition fragments. As a result, adenine and uridine additions account for almost 80% of all addition events. Only the addition fragments accounting for more than 1% of all addition events are listed here.

Sequence variations at the 3' ends have been often observed in miRNA reads. Previous studies also reported several types of RNA editings, such as A to G transition catalyzed by adenosine deaminase and C to U transition catalyzed by cytidine deaminase [12,19], responsible for generating such variations. Owing to the existence of isomiR, it is difficult to distinguish whether nucleotide addition or nucleotide modification contributes to such variations. As illustrated in Additional file 4, the terminal nucleotide variation, also called mismatch, could be generated from nucleotide modification from C to U at the terminus of the sequence read with 22 nucleotides, altering the length of the read. Alternatively, it could also be generated from nucleotide addition of U to the terminus of the read with 21 nucleotides, lengthening the read by one nucleotide. Additional molecular studies would be required to elucidate specific mechanism involved.

### Inconsistent expression ratios of 5p arm to 3p arm

According to the hydrogen-bonding theory, the selection preference between pre-miRNA's 5p arm and 3p arm is an intrinsic characteristic of pre-miRNA. However, if this selection theory is the only criterion deciding arm selection preference, the expression ratio of 5p arm to 3p arm should be consistent, with only slight difference, wherever the sequencing samples come from. In order to examine whether the expression ratio for each pre-miRNA is consistent or not, we investigated its expression ratios of 5p arm to 3p arm in G1245N library and in G1245T library, naming them Ratio_N53p and Ratio_T53p, respectively. As shown in Table 3, the 5p and 3p miRNA of hsa-mir-423 individually have read counts of 20,920 (N5p) and 8,945 (N3p) in G1245N library, leading to the Ratio_N53p value of 2.3387. However, the Ratio_T53p value in G1245T library is 0.2726, leading to 8.58 fold change between Ratio_N53p to Ratio_T53p. In order to avoid dramatic change in expression ratio of 5p arm to 3p arm, we examined only 166 pre-miRNAs whose N5p, N3p, T5p and T3p are equal to or larger than 10.

**Table 3:** Inconsistent expression ratios of 5p arm miRNA to 3p arm miRNA between tissues

Pre-miRNA	Location	N5p	N3p	Ratio_N53p	T5p	T3p	Ratio_T53p	Fold change
hsa-mir-17	MA:14-36;mi:51-72	4,767	2,266	2.1037	6,656	425	15.6612	7.44
hsa-mir-511-1	5p:16-36	39	903	0.0432	53	147	0.3605	8.35
hsa-mir-511-2	5p:16-36	39	903	0.0432	53	147	0.3605	8.35
hsa-mir-423	5p:17-39;3p:53-75	20,920	8,945	2.3387	1,708	6,266	0.2726	8.58
hsa-mir-30c-1	MA:17-39;mi:56-77	67,040	50	1,340.8000	8,685	64	135.7031	9.88
hsa-mir-135b	MA:16-38;mi:55-76	360	19	18.9474	171	99	1.7273	10.97
hsa-mir-376a-1	mi:7-28;MA:44-64	1,646	14	117.5714	569	54	10.5370	11.16
hsa-mir-376a-2	3p:50-70	428	14	30.5714	99	54	1.8333	16.68
hsa-mir-335	MA:16-38;mi:52-73	3,509	110	31.9000	418	264	1.5833	20.15
hsa-mir-30b	MA:17-38;mi:55-76	50,988	51	999.7647	3,931	89	44.1685	22.64

Location column denotes the relative locations of mature miRNAs within their precursors. N5p, T5p individually denote the expression levels of 5p arm miRNA (determined by summing the read counts of all its isomiRs) in normal and tumor tissue; while, N3p, T3p denote the corresponding information of 3p arm miRNA. The N5p and N3p values shown in this study are normalized.

**Figure 3:**
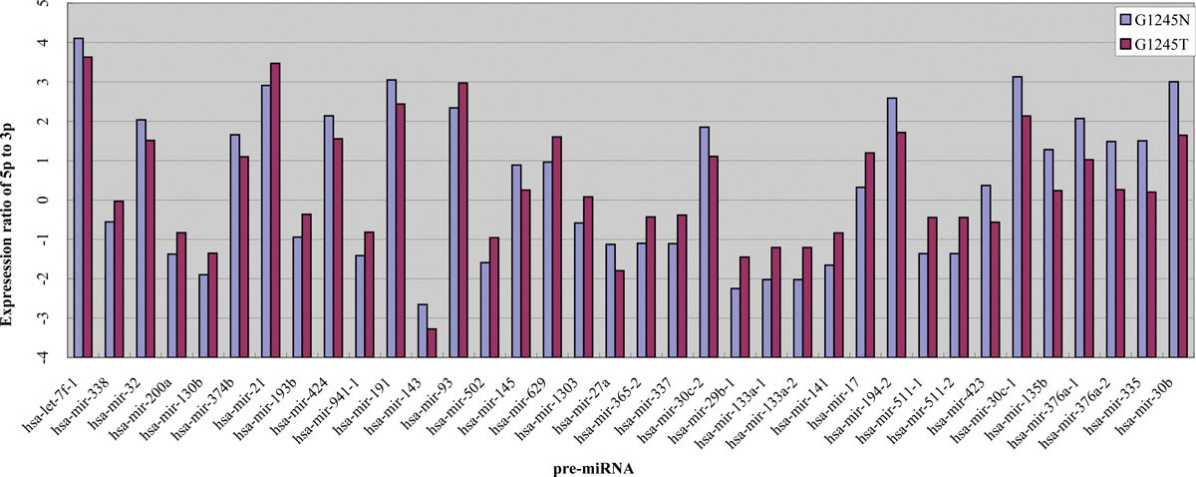
**The expression ratios of 5p arm miRNA to 3p arm miRNA.** Several pre-miRNAs have significantly different ratios, demonstrating the arm selection preferences within the pre-miRNAs are significantly different between gastric normal and gastric tumor tissue.

By an empirical criterion of fold change difference larger than three, 36 out of 166 examined pre-miRNAs show different expression ratios of 5p to 3p in normal and tumor gastric tissue. As shown in Figure 3, several pre-miRNAs have significantly different ratios, demonstrating that the arm selection preferences within the pre-miRNAs are significantly different between gastric normal and gastric tumor tissue. For more detailed examination, we listed the information of the 36 significant pre-miRNAs in Table 3 and Additional file 5. As illustrated in Table 3, the fold change can even reach up to more than 20. Among the significant altered pre-miRNAs, hsa-mir-17 is a very interesting case, at which 5p arm has almost two times higher expression level than 3p arm in normal tissue. However, in tumor tissue, the 5p arm becomes about 1.4 times higher than in normal tissue but the 3p arm becomes 5.3 times lower, which results in the large difference in expression ratio of 5p to 3p. Even with large difference in expression ratio of 5p to 3p, the major arm of hsa-mir-17 in normal and tumor tissue is the same one, the 5p arm. In another interesting case of hsa-mir-423, the major arm and minor arm in normal tissue are individually 5p arm and 3p arm, obeying the annotation of miRBase. However, in tumor tissue, the major arm and minor arm are individually the 3p arm and 5p arm, totally reverse to miRBase annotation. In this case, the preferential arm in normal and tumor tissue shift from 5p to 3p.

In summary, our result showed the 5p and 3p preference is not always consistent between biological samples, implying there could be other regulation mechanisms, in addition to the hydrogen-bonding-based selection rule, controlling the selection of 5p or 3p. If so, this regulation mechanism could play important roles in oncogenesis process. This is a novel area to study the relationship between miRNAs and cancers. Therefore, more efforts should be applied in the subsequent studies.

### Arm selection preference between normal and tumor tissue

In our previous study, we have shown arm selection preference of the same pre-miRNAs could vary between species [9]. In this study, our result implies there could be regulation mechanism, in addition to the hydrogen-bonding-based selection rule, controlling the selection of 5p arm or 3p arm between different tissues and samples. Therefore, we are curious about whether difference on arm selection preference can be observed between tissues, especially between normal and tumor tissue. We first examined whether the arm selection preference annotated by miRBase is consistent with our NGS expression data. As shown in Table 4, the arm selection preferences of 22 pre-miRNAs are opposite to miRBase annotation. According to miRBase annotation, hsa-mir-1277, hsa-mir-376a-2, hsa-mir-495, hsa-mir-659, hsa-mir-1303, hsa-mir-1307 and so on encode mature miRNA at only their 3p arms. We not only detected mature miRNA at their 5p arms but also observed that the newly detected 5p arms have higher expression levels than the originally annotated 3p arm. Besides, the newly detected 3p arms of hsa-mir-511-1, hsa-mir-511-2, hsa-mir-1273c and hsa-mir-1247 also have higher expression levels than the originally annotated 5p arm.

**Table 4:** The pre-miRNAs whose arm selection preferences are not consistent with miRBase annotation

pre-miRNA	Location	N5p	N3p	T5p	T3p	MAmiExch
hsa-mir-1277	3p:47-68	354	172	201	56	N;T
hsa-mir-376a-2	3p:50-70	428	14	99	54	N;T
hsa-mir-495	3p:50-71	35	19	12	5	N;T
hsa-mir-1303	3p:52-73	19	73	12	10	T
hsa-mir-1306	3p:55-72	94	0	31	0	N;T
hsa-mir-496	3p:56-77	27	0	50	0	N;T
hsa-mir-561	3p:61-82	231	0	40	0	N;T
hsa-mir-659	3p:61-82	123	0	56	0	N;T
hsa-mir-376b	3p:62-83	106	9	32	83	N
hsa-mir-1307	3p:80-101	3512	2369	2002	3544	N
hsa-mir-1273c	5p:10-31	0	53	0	50	N;T
hsa-mir-511-1	5p:16-36	39	903	53	147	N;T
hsa-mir-511-2	5p:16-36	39	903	53	147	N;T
hsa-mir-1247	5p:40-61	67	237	17	41	N;T
hsa-mir-374a	MA:12-33;mi:42-63	2275	36122	2017	35147	N;T
hsa-mir-500a	MA:13-35;mi:52-73	215	1907	72	1834	N;T
hsa-mir-625	MA:15-35;mi:52-73	333	576	87	206	N;T
hsa-mir-136	MA:15-37;mi:49-70	1328	621	530	652	T
hsa-mir-664	mi:11-34;MA:49-71	913	271	398	98	N;T
hsa-mir-144	mi:15-36;MA:52-71	12406	441	1209	113	N;T
hsa-mir-493	mi:16-37;MA:57-78	254	248	1744	727	N;T
hsa-mir-376a-1	mi:7-28;MA:44-64	1646	14	569	54	N;T

N and T symbols in "MAmiExch" column represent the normal and tumor library in which such major and minor arm exchange event was observed.

For the pre-miRNAs originally annotated to encode miRNAs at both arms, the major arms of hsa-mir-374a, hsa-mir-500a, hsa-mir-625 and hsa-mir-136 are their 5p arms; while, the major arms of hsa-mir-664, hsa-mir-144, hsa-mir-493 and hsa-mir-376a-1 are their 3p arms. According to NGS expression data, we observed that their major arms and minor arms expression levels reversed, leading to an observation different from miRBase annotation. Among them, hsa-mir-374a and hsa-mir-144 are two extreme cases, at which the miRBase-annotated minor arms individually have about 17 or 28 times as high expression levels as the miRBase-annotated major arms have. In summary, our result demonstrated that arm selection preference could vary. In order to solve this debate, more NGS expression data should be included and such phenomenon should be studied further.

Since arm selection preference is inconsistent between known reported miRNAs and our NGS expression data, we further investigated whether arm selection preference could vary between normal and tumor tissue. For this purpose, we examined whether arm selection preference differs between normal and tumor tissue. In other words, we investigated whether the 5p arm and 3p arm miRNA derived from the same pre-miRNA have reversal tissue expression preference without considering miRBase annotation. As shown in Table 5, we observed four additional pre-miRNAs possessing this property. Within hsa-mir-136, hsa-mir-423, hsa-mir-376b and hsa-mir-1307, their 5p arm miRNAs have higher expression level in the normal tissue; while their 3p arm miRNAs dominated in the tumor tissue. In the contrary, the 5p arm of hsa-mir-361 is the major miRNA in tumor tissue and the 3p arm miRNA is the major one in normal tissue.

**Table 5:** Arm selection preference of 5p arm and 3p arm miRNA exchange between gastric normal and gastric tumor tissue

pre-miRNA	Location	N5p	N3p	T5p	T3p
hsa-mir-136	MA:15-37;mi:49-70	1328	621	530	652
hsa-mir-361	5p:6-27;3p:45-67	1651	2493	1451	1024
hsa-mir-423	5p:17-39;3p:53-75	20920	8945	1708	6266
hsa-mir-376b	3p:62-83	106	9	32	83
hsa-mir-1307	3p:80-101	3512	2369	2002	3544

In this table, the major and minor mature miRNA dereived from the same pre-miRNA exchange between gastric normal and gastric tumor tissue.

Although derived from the same gene locus and transcribed by the same transcription factors, the 5p arm and 3p arm miRNA might have reversal expression preference. It is likely that such an observation is generated by NGS platform dependent biases. However, it is possible that there could be an unknown selection mechanism, during maturation procedure, controlling the arm selection preference between normal and tumor tissue. According to our data, this idea is reasonable and deserves more efforts to put it into examination.

### Systematic investigation on isomiR distribution between tissues

Previous report showed that different isomiR types may contribute to different regulation in different tissues [13]. In this study, we are curious about whether isomiR distribution patterns are diverse between gastric normal and gastric tumor tissue, namely normal tissue prefers several specific isomiR types and tumor prefers the others. In order to solve this problem, we applied Kolmogorov-Smirnov (KS) test in determining significant difference on isomiR distribution patterns. KS test tries to determine if two datasets differ significantly under the null hypothesis that the samples are drawn from the same distribution. Although KS test has the advantage of making no assumption about the distribution of data, it is sensitive to the median. Besides, when a miRNA has significant low expression level, only few isomiR types can be presented, inducing biased isomiR distribution patterns. Therefore, we selected only the 169 miRNAs with more than eight kinds of isomiR types and read counts more than 1000 in both normal and tumor tissue for comparison.

**Figure 4:**
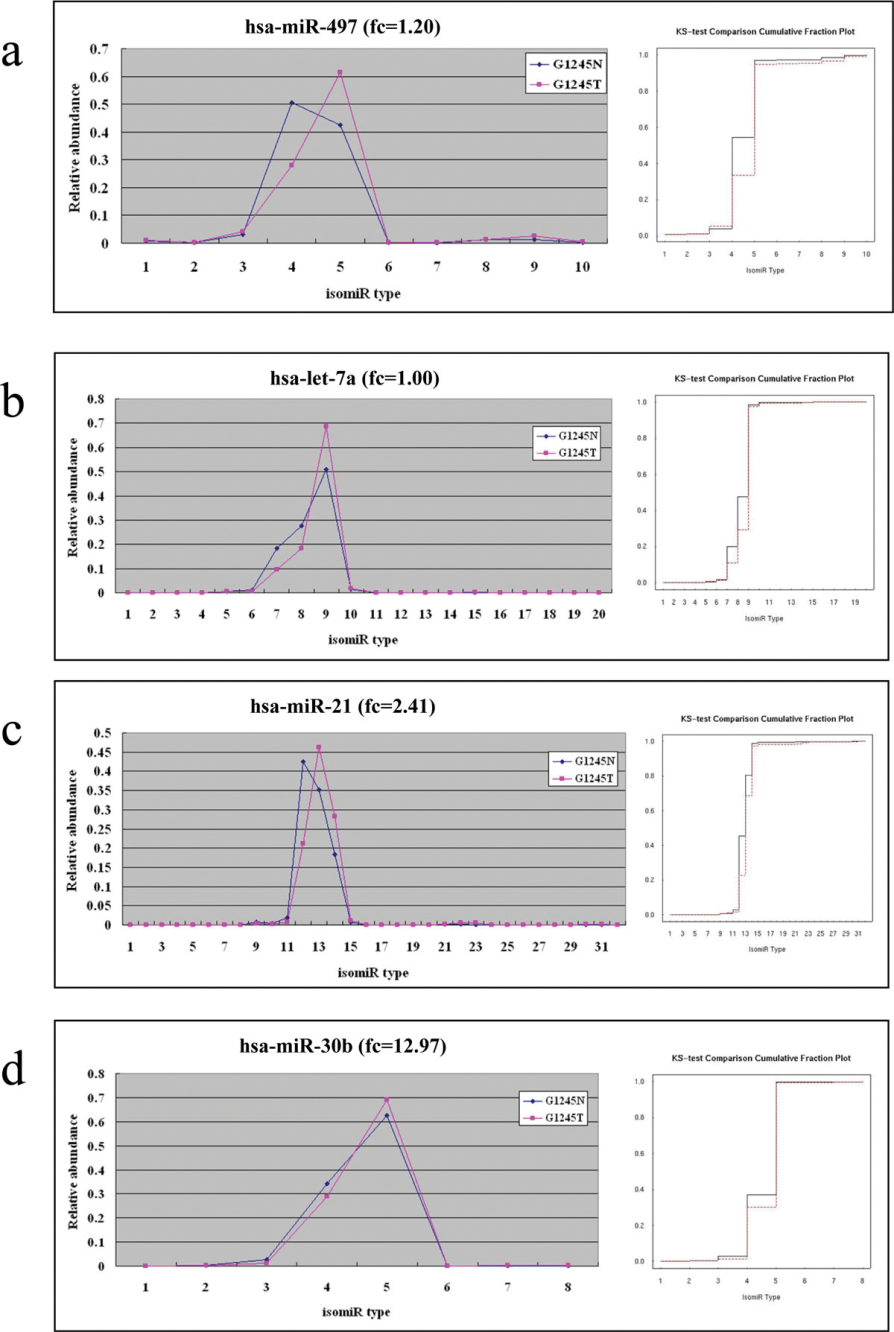
**IsomiR distribution patterns mature miRNAs between G1245N and G1245T library.** The isomiR distribution patterns (left panel) and the corresponding KS-test comparison plot (right panel) are provided. (a) The most abundant isomiRs in two tissues are type four and type five, respectively. (b) The most abundant isomiR type in G1245T is 20% less in G1245N. (c) The most abundant isomiRs in two tissues are individually type 12 and type 13. (d) Even expression levels of hsa-miR-30b are dramatically different between tissues, the isomiR distribution patterns are still the same.

Under our p value criterion < 0.001 (see Methods), 42 out of the 169 examined miRNAs are identified to have different isomiR distribution patterns between normal and tumor gastric tissue. The detailed information of the significant miRNAs is listed in Additional file 6. Below, we illustrated several cases for discussion. As illustrated in Figure 4a, the expression levels of hsa-miR-497 are approximately equal in both tissues; while, it has diverse isomiR distribution patterns (p value≈0) between G1235N and G1245T tissue. In G1245N, the most and secondary abundant isomiRs are type four and type five. In G1245T, isomiR type four and type five reversed in terms of abundance. In Figure 4b, although the isomiR preferences of hsa-let-7a between tissues are the same, the most abundant isomiR type in G1245T is 20% less in G1245N. Moreover, both the secondary and tertiary abundant isomiR type in G1245N increase 10% than that in G1245T.

For the previous two cases, the expression levels between two tissues are almost equal. In Figure 4c, the expression levels of hsa-miR-21 are different between tissues, with fold change (fc) equal to 2.4051. In this case, the isomiR distribution patterns are diverse. The isomiR type peaks shift and both the most and secondary abundant isomiR type between tissues are different. So far, we have shown no matter the fc value is small or large, the isomiR distribution type can be diverse between tissues. Next, in Figure 4d, even the fc value of hsa-miR-30b reaches up to 12.97, the isomiR distribution patterns are not diverse between tissues.

Although isomiRs are highly overlapped with each other, they could have difference at the 5' end, leading to alteration at the seed region. The complementary binding between miRNAs and target mRNAs mainly depends on the binding within the seed region. Therefore, the difference at the 5'end between isomiRs is supposed to alter isomiRs' target genes. As a result, different isomiRs from the miRNAs could have different target genes, involved in different activities or pathways. Hence, it is reasonable that different isomiRs may contribute to various regulation pathways in different tissues. And, this type of isomiR regulation could have significant biological consequences.

## Conclusions

In this study, we applied NGS data to quantify miRNA expression profiles between gastric normal and gastric tumor tissue. Our data showed that although derived from the same pre-miRNAs, 5p arm miRNA and 3p arm miRNA can have reversed expression preferences, implying there could be other regulation mechanism controlling 5p or 3p selection. Moreover, although derived from the same mature miRNA, isomiRs can have different expression preference, some prefer normal tissue and the other prefer tumor tissue. Although we examined only one pair of normal and tumor tissue, our results provided a novel research topic in miRNA regulation study. With more tissue samples examined, we can have more robust conclusions and perform the studies with insight into miRNA regulation.

## Abbreviations

microRNA, NGS, isomiR, gastric tumor, arm selection.

## Competing interests

The authors declare that they have no competing interests.

## Authors' contributions

SCL executed this study and wrote the draft of this manuscript. YLL is responsible for PCR validation of miRNA. MRH helped calculate the p value of KS test. KWT and CHL helped tissue preparation and RNA extraction. WCL supervised the study and edited the manuscript.

## Supplementary Material

Additional file 1 **The criteria of our mapping procedure.** In order to exclude random match, the difference in start position between mature miRNA and mapped reads must be equal to or less than two. While, the difference in end position between mature miRNA and mapped reads must be equal to or less than five. Click here for file

Additional file 2 **The mapping result of all pre-miRNAs in G1245N library.** We arranged the miRNA reads from G1245N library within the mapped pre-miRNAs. The read count and location offset of each isomiR are provided. Click here for file

Additional file 3 **The mapping result of all pre-miRNAs in G1245N library.** We arranged the miRNA reads from G1245T library within the mapped pre-miRNAs. The read count and location offset of each isomiR are provided. Click here for file

Additional file 4 **The difficulty in distinguishing nucleotide addition from nucleotide modification.** In this case, the last nucleotide variation could be generated from nucleotide modification from C to U at the terminus of the sequence read with 22 nucleotides, which does not alter the length of the read. However, it could also be generated from nucleotide addition of U to the terminus of the read with 21 nucleotides, which lengthens the read by one nucleotide. Click here for file

Additional file 5 **Inconsistent expression ratios of 5p arm miRNA to 3p arm miRNA.** The expression ratios of 5p arm miRNA to 3p arm miRNA are not consistent between gastric normal and gastric tumor tissue, which implies the arm selection preference of 5p arm and 3p arm may varyn between tissues. Click here for file

Additional file 6 **List of miRNAs whose isomiR distribution patterns significantly differ between normal and tumor tissue.** Normal and Tumor denote the expression level of mature miRNA in gastric normal and gastric tumor tissue, respectively. Fold change denote the fold change of miRNA expression level. Click here for file
