# Supplementary figures and images for: miRNA arm selection and isomiR distribution in gastric cancer
Source: BMC Genomics. 2012 Jan 17;13(Suppl 1):S13. doi: 10.1186/1471-2164-13-S1-S13 (PMC3303722; doi:10.1186/1471-2164-13-S1-S13)

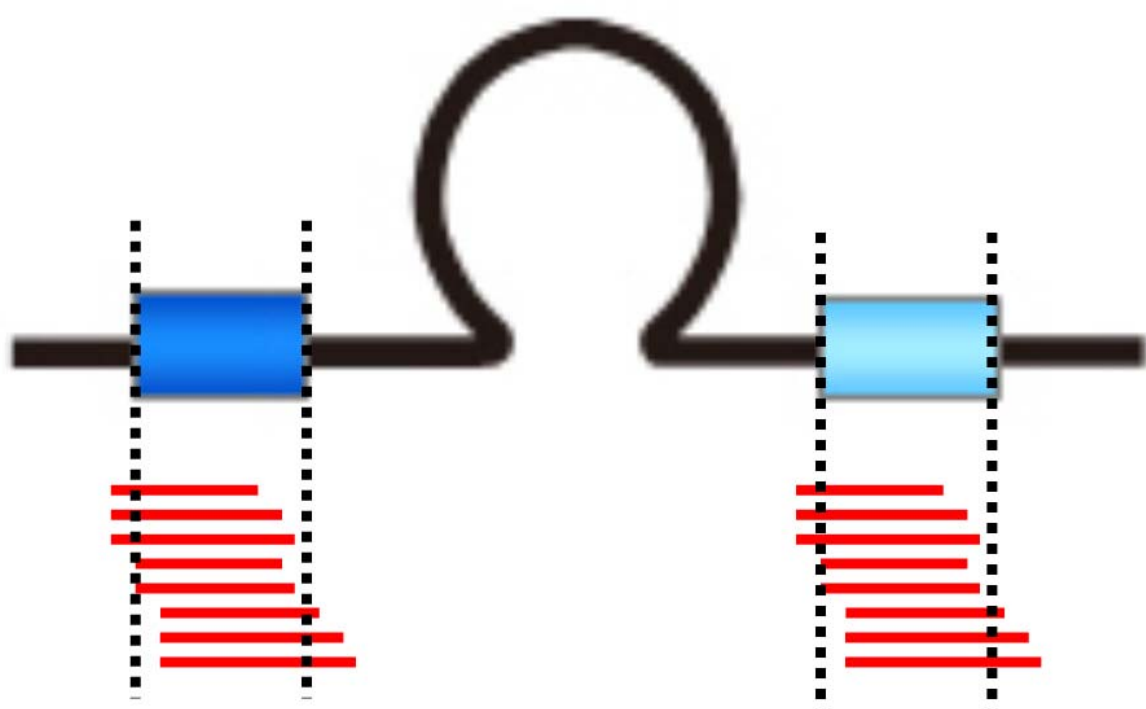

Supplement: Additional file 1 — The criteria of our mapping procedure. In order to exclude random match, the difference in start position between mature miRNA and mapped reads must be equal to or less than two. While, the difference in end position between mature miRNA and mapped reads must be equal to or less than five. [file 1471-2164-13-S1-S13-S1.pdf]

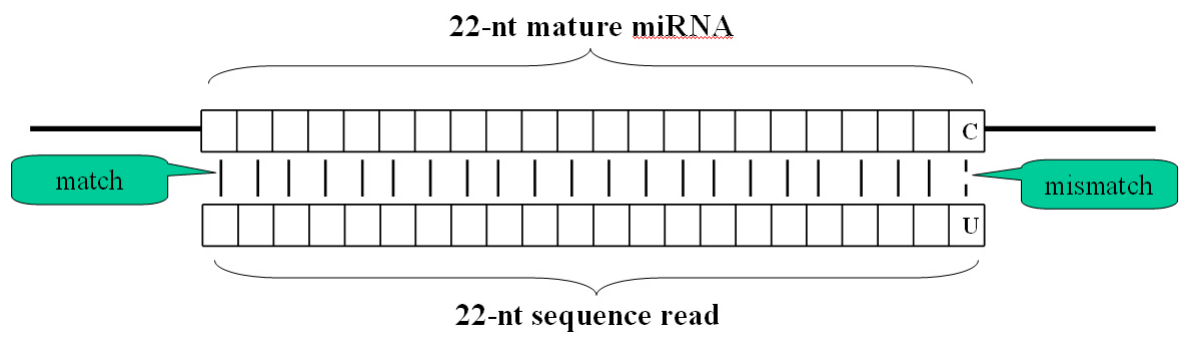

Supplement: Additional file 4 — The difficulty in distinguishing nucleotide addition from nucleotide modification. In this case, the last nucleotide variation could be generated from nucleotide modification from C to U at the terminus of the sequence read with 22 nucleotides, which does not alter the length of the read. However, it could also be generated from nucleotide addition of U to the terminus of the read with 21 nucleotides, which lengthens the read by one nucleotide. [file 1471-2164-13-S1-S13-S4.pdf]
