# Supplementary material for: miRNA arm selection and isomiR distribution in gastric cancer
Source: BMC Genomics. 2012 Jan 17;13(Suppl 1):S13. doi: 10.1186/1471-2164-13-S1-S13 (PMC3303722; doi:10.1186/1471-2164-13-S1-S13)
Supplement: Additional file 5 — Inconsistent expression ratios of 5p arm miRNA to 3p arm miRNA. The expression ratios of 5p arm miRNA to 3p arm miRNA are not consistent between gastric normal and gastric tumor tissue, which implies the arm selection preference of 5p arm and 3p arm may varyn between tissues. [file 1471-2164-13-S1-S13-S5.doc]

**Additional file 5. Inconsistent expression ratios of 5p arm miRNA to 3p arm miRNA.** The expression ratios of 5p arm miRNA to 3p arm miRNA are not consistent between gastric normal and gastric tumor tissue, which implies the arm selection preference of 5p arm and 3p arm may varyn between tissues.

| **Pre-miRNA** | **Presentation** | **N5p** | **N3p** | **Ratio_N53p** | **T5p** | **T3p** | **Ratio_T53p** | **Fold change** |
| --- | --- | --- | --- | --- | --- | --- | --- | --- |
| hsa-mir-511-1 | 5pM:16-36 | 39 | 903 | 0.0432 | 53 | 147 | 0.3605 | 8.35 |
| hsa-mir-511-2 | 5pM:16-36 | 39 | 903 | 0.0432 | 53 | 147 | 0.3605 | 8.35 |
| hsa-mir-17 | MA:14-36;mi:51-72 | 4,767 | 2,266 | 2.1037 | 6,656 | 425 | 15.6612 | 7.44 |
| hsa-mir-141 | mi:17-38;MA:59-80 | 3,504 | 158,383 | 0.0221 | 1,097 | 7,480 | 0.1467 | 6.63 |
| hsa-mir-133a-1 | 3pM:53-74 | 11 | 1,165 | 0.0094 | 30 | 485 | 0.0619 | 6.55 |
| hsa-mir-133a-2 | 3pM:59-80 | 11 | 1,165 | 0.0094 | 30 | 485 | 0.0619 | 6.55 |
| hsa-mir-29b-1 | mi:10-33;MA:51-73 | 168 | 29,985 | 0.0056 | 298 | 8,434 | 0.0353 | 6.31 |
| hsa-mir-337 | 5p:23-43;3p:61-82 | 22 | 285 | 0.0772 | 52 | 125 | 0.4160 | 5.39 |
| hsa-mir-365-2 | mi:29-50;MA:68-89 | 23 | 291 | 0.0790 | 46 | 124 | 0.3710 | 4.69 |
| hsa-mir-1303 | 3pM:52-73 | 19 | 73 | 0.2603 | 12 | 10 | 1.2000 | 4.61 |
| hsa-mir-629 | MA:22-42;mi:61-82 | 1,315 | 143 | 9.1958 | 723 | 18 | 40.1667 | 4.37 |
| hsa-mir-502 | 5p:16-36;3p:52-73 | 26 | 1,012 | 0.0257 | 39 | 352 | 0.1108 | 4.31 |
| hsa-mir-93 | MA:11-33;mi:50-71 | 21,248 | 97 | 219.0515 | 94,118 | 101 | 931.8614 | 4.25 |
| hsa-mir-941-1 | 3pM:71-93 | 10 | 258 | 0.0388 | 26 | 171 | 0.1520 | 3.92 |
| hsa-mir-193b | mi:14-35;MA:51-72 | 222 | 1,937 | 0.1146 | 69 | 160 | 0.4313 | 3.76 |
| hsa-mir-21 | MA:8-29;mi:46-66 | 2,904,440 | 3,590 | 809.0362 | 6,985,499 | 2,367 | 2,951.2036 | 3.65 |
| hsa-mir-130b | mi:13-33;MA:51-72 | 138 | 10,939 | 0.0126 | 55 | 1,243 | 0.0442 | 3.51 |
| hsa-mir-200a | mi:16-37;MA:54-75 | 4,080 | 96,911 | 0.0421 | 1,565 | 10,599 | 0.1477 | 3.51 |
| hsa-mir-338 | 5p:6-27;3p:42-63 | 1,118 | 4,008 | 0.2789 | 513 | 553 | 0.9277 | 3.33 |
| hsa-let-7f-1 | MA:7-28;mi:63-84 | 814,310 | 64 | 12,723.5938 | 368,468 | 87 | 4,235.2644 | 3.00 |
| hsa-mir-32 | MA:6-27;mi:47-68 | 2,390 | 22 | 108.6364 | 848 | 26 | 32.6154 | 3.33 |
| hsa-mir-374b | MA:11-32;mi:41-62 | 3,039 | 67 | 45.3582 | 1,613 | 129 | 12.5039 | 3.63 |
| hsa-mir-424 | MA:11-32;mi:48-68 | 8,360 | 61 | 137.0492 | 3,077 | 86 | 35.7791 | 3.83 |
| hsa-mir-191 | MA:16-38;mi:58-79 | 77,535 | 69 | 1,123.6957 | 22,826 | 83 | 275.0120 | 4.09 |
| hsa-mir-143 | mi:27-48;MA:61-81 | 6,070 | 2,748,993 | 0.0022 | 4,506 | 8,516,990 | 0.0005 | 4.17 |
| hsa-mir-145 | MA:16-38;mi:54-75 | 31,233 | 4,037 | 7.7367 | 13,568 | 7,601 | 1.7850 | 4.33 |
| hsa-mir-27a | mi:10-31;MA:51-71 | 1,195 | 16,051 | 0.0745 | 549 | 34,213 | 0.0160 | 4.64 |
| hsa-mir-30c-2 | MA:7-29;mi:47-68 | 67,053 | 953 | 70.3599 | 8,685 | 682 | 12.7346 | 5.53 |
| hsa-mir-194-2 | MA:15-36;mi:51-72 | 246,950 | 640 | 385.8594 | 38,304 | 744 | 51.4839 | 7.49 |
| hsa-mir-423 | 5p:17-39;3p:53-75 | 20,920 | 8,945 | 2.3387 | 1,708 | 6,266 | 0.2726 | 8.58 |
| hsa-mir-30c-1 | MA:17-39;mi:56-77 | 67,040 | 50 | 1,340.8000 | 8,685 | 64 | 135.7031 | 9.88 |
| hsa-mir-135b | MA:16-38;mi:55-76 | 360 | 19 | 18.9474 | 171 | 99 | 1.7273 | 10.97 |
| hsa-mir-376a-1 | mi:7-28;MA:44-64 | 1,646 | 14 | 117.5714 | 569 | 54 | 10.5370 | 11.16 |
| hsa-mir-376a-2 | 3pM:50-70 | 428 | 14 | 30.5714 | 99 | 54 | 1.8333 | 16.68 |
| hsa-mir-335 | MA:16-38;mi:52-73 | 3,509 | 110 | 31.9000 | 418 | 264 | 1.5833 | 20.15 |
| hsa-mir-30b | MA:17-38;mi:55-76 | 50,988 | 51 | 999.7647 | 3,931 | 89 | 44.1685 | 22.64 |
