# Supplementary material for: miRNA arm selection and isomiR distribution in gastric cancer
Source: BMC Genomics. 2012 Jan 17;13(Suppl 1):S13. doi: 10.1186/1471-2164-13-S1-S13 (PMC3303722; doi:10.1186/1471-2164-13-S1-S13)
Supplement: Additional file 6 — List of miRNAs whose isomiR distribution patterns significantly differ between normal and tumor tissue. Normal and Tumor denote the expression level of mature miRNA in gastric normal and gastric tumor tissue, respectively. Fold change denote the fold change of miRNA expression level. [file 1471-2164-13-S1-S13-S6.doc]

**Additional file 6. List of miRNAs whose isomiR distribution patterns significantly differ between normal and tumor tissue.** Normal and Tumor denote the expression level of mature miRNA in gastric normal and gastric tumor tissue, respectively. Fold change denote the fold change of miRNA expression level.

| Mature miRNA | pre-miRNA | Normal | Tumor | Fold change | p_value |
| --- | --- | --- | --- | --- | --- |
| hsa-let-7a | hsa-let-7a-1 | 1056315 | 1059110 | 1.0026 | 0 |
| hsa-let-7a | hsa-let-7a-2 | 1056201 | 1059186 | 1.0028 | 0 |
| hsa-let-7a | hsa-let-7a-3 | 1058723 | 1064782 | 1.0057 | 0 |
| hsa-let-7b | hsa-let-7b | 736529 | 809249 | 1.0987 | 0 |
| hsa-let-7f | hsa-let-7f-1 | 814310 | 368468 | 2.2099 | 0 |
| hsa-let-7f | hsa-let-7f-2 | 821458 | 372323 | 2.2063 | 0 |
| hsa-miR-106b* | hsa-mir-106b | 2038 | 3186 | 1.5632 | 2.89E-15 |
| hsa-miR-107 | hsa-mir-107 | 2195 | 1425 | 1.5403 | 2.71E-08 |
| hsa-miR-1 | hsa-mir-1-1 | 60741 | 324757 | 5.3465 | 0 |
| hsa-miR-1307 | hsa-mir-1307 | 2369 | 3544 | 1.4959 | 9.57E-14 |
| hsa-mir-1307-opp ^#^ | hsa-mir-1307 | 3512 | 2002 | 1.7542 | 2.12E-06 |
| hsa-miR-130b | hsa-mir-130b | 10939 | 1243 | 8.8004 | 0 |
| hsa-miR-143 | hsa-mir-143 | 2748993 | 8516990 | 3.0982 | 0 |
| hsa-miR-148a | hsa-mir-148a | 3799531 | 202732 | 18.741 | 0 |
| hsa-miR-150 | hsa-mir-150 | 4288 | 1971 | 2.1755 | 3.42E-08 |
| hsa-miR-15b | hsa-mir-15b | 3628 | 1754 | 2.0684 | 3.33E-15 |
| hsa-miR-18a | hsa-mir-18a | 1618 | 1655 | 1.0228 | 1.33E-15 |
| hsa-miR-190 | hsa-mir-190 | 5512 | 1469 | 3.7522 | 0 |
| hsa-miR-192 | hsa-mir-192 | 2325366 | 424939 | 5.4722 | 0 |
| hsa-miR-199a-5p | hsa-mir-199a-2 | 2391 | 9060 | 3.7892 | 0.000204993 |
| hsa-miR-19b | hsa-mir-19b-1 | 5709 | 1577 | 3.6201 | 1.33E-15 |
| hsa-miR-19b | hsa-mir-19b-2 | 5709 | 1577 | 3.6201 | 1.33E-15 |
| hsa-miR-21 | hsa-mir-21 | 2904440 | 6985499 | 2.4051 | 0 |
| hsa-miR-222 | hsa-mir-222 | 3239 | 1398 | 2.3168 | 7.50E-10 |
| hsa-miR-223 | hsa-mir-223 | 2013 | 1221 | 1.6486 | 3.55E-05 |
| hsa-miR-27b* | hsa-mir-27b | 1348 | 2162 | 1.6038 | 6.66E-16 |
| hsa-miR-27b | hsa-mir-27b | 82575 | 176023 | 2.1316 | 3.33E-16 |
| hsa-miR-339-5p | hsa-mir-339 | 6486 | 1462 | 4.4363 | 0 |
| hsa-miR-34a | hsa-mir-34a | 3205 | 1861 | 1.7221 | 1.75E-11 |
| hsa-miR-361-5p | hsa-mir-361 | 1651 | 1451 | 1.1378 | 2.50E-11 |
| hsa-miR-361-3p | hsa-mir-361 | 2493 | 1024 | 2.4345 | 0 |
| hsa-miR-375 | hsa-mir-375 | 490533 | 7462 | 65.737 | 0 |
| hsa-miR-378 | hsa-mir-378 | 1282842 | 252543 | 5.0796 | 0 |
| hsa-miR-423-5p | hsa-mir-423 | 20920 | 1708 | 12.248 | 1.19E-09 |
| hsa-miR-452 | hsa-mir-452 | 1472 | 3357 | 2.2805 | 0 |
| hsa-miR-497 | hsa-mir-497 | 1671 | 1391 | 1.2012 | 0 |
| hsa-miR-500a | hsa-mir-500a | 1907 | 1834 | 1.0398 | 1.11E-16 |
| hsa-miR-652 | hsa-mir-652 | 3276 | 1729 | 1.8947 | 0.000606345 |
| hsa-miR-671-5p | hsa-mir-671 | 4040 | 1558 | 2.593 | 0 |
| hsa-miR-744 | hsa-mir-744 | 1501 | 2425 | 1.6155 | 4.80E-14 |
| hsa-miR-769-5p | hsa-mir-769 | 3101 | 1770 | 1.7519 | 0.000436954 |
| hsa-miR-96 | hsa-mir-96 | 7663 | 1007 | 7.6097 | 2.89E-15 |

^#^ jhadjdad
